# Pdro, a Protein Associated with Late Endosomes and Lysosomes and Implicated in Cellular Cholesterol Homeostasis

**DOI:** 10.1371/journal.pone.0010977

**Published:** 2010-06-08

**Authors:** Patricia Guillaumot, Céline Luquain, Mouhannad Malek, Anne-Laure Huber, Sabine Brugière, Jérome Garin, Didier Grunwald, Daniel Régnier, Virginie Pétrilli, Etienne Lefai, Serge N. Manié

**Affiliations:** 1 Génétique Moléculaire, Signalisation et Cancer, UMR 5201 CNRS, Centre Leon Berard, Lyon, France; 2 Regulation Métabolique, Nutrition et Diabète, UMR 870 INSERM/Insa-Lyon, Villeurbanne, France; 3 Laboratoire de Chimie des Protéines, ERM 201 INSERM/CEA/UJF, CEA/Grenoble, Grenoble, France; 4 Laboratoire Transduction de Signal, Unité 873, INSERM/CEA/DSV, Institut de Recherches en Technologies et Sciences pour le Vivant, Grenoble, France; 5 INRA, UMR 1235, INSERM, U870, Oullins, France; University of Nebraska Medical Center, United States of America

## Abstract

**Background:**

Cellular cholesterol is a vital component of the cell membrane. Its concentration is tightly controlled by mechanisms that remain only partially characterized. In this study, we describe a late endosome/lysosomes–associated protein whose expression level affects cellular free cholesterol content.

**Methodology/Principal Findings:**

Using a restricted proteomic analysis of detergent-resistant membranes (DRMs), we have identified a protein encoded by gene C11orf59. It is mainly localized to late endosome/lysosome (LE/LY) compartment through N-terminal myristoylation and palmitoylation. We named it Pdro for protein associated with DRMs and endosomes. Very recently, three studies have reported on the same protein under two other names: the human p27RF-Rho that regulates RhoA activation and actin dynamics, and its rodent orthologue p18 that controls both LE/LY dynamics through the MERK-ERK pathway and the lysosomal activation of mammalian target of rapamycin complex 1 by amino acids. We found that, consistent with the presence of sterol-responsive element consensus sequences in the promoter region of C11orf59, Pdro mRNA and protein expression levels are regulated positively by cellular cholesterol depletion and negatively by cellular cholesterol loading. Conversely, Pdro is involved in the regulation of cholesterol homeostasis, since its depletion by siRNA increases cellular free cholesterol content that is accompanied by an increased cholesterol efflux from cells. On the other hand, cells stably overexpressing Pdro display reduced cellular free cholesterol content. Pdro depletion-mediated excess cholesterol results, at least in part, from a stimulated low-density lipoprotein (LDL) uptake and an increased cholesterol egress from LE/LY.

**Conclusions/Significance:**

LDL-derived cholesterol release involves LE/LY motility that is linked to actin dynamics. Because Pdro regulates these two processes, we propose that modulation of Pdro expression in response to sterol levels regulates LDL-derived cholesterol through both LDL uptake and LE/LY dynamics, to ultimately control free cholesterol homeostasis.

## Introduction

Cholesterol is essential for maintenance of membrane integrity and multiple cellular functions. However, excess cholesterol is toxic and therefore cells maintain their concentration of cholesterol under tight control [1], [2]. Mammalian cells growing under ordinary culture conditions derive their cholesterol preferentially from endocytic uptake of low-density lipoproteins (LDL) present in the serum of the culture medium, and *de novo* synthesis in the endoplasmic reticulum (ER) is usually kept suppressed. Internalized lipoprotein-associated cholesterol esters are hydrolyzed to free cholesterol in late endosome/lysosome (LE/LY), from which it is exported to various destinations, including the plasma membrane and the endoplasmic reticulum. Precisely how cholesterol egresses from LE/LY remains incompletely characterized. The Niemman-Pick Type C (NPC) disease, an inherited lipid storage disorder, is a well-known example of free cholesterol accumulation in LE/LY [1]. As a result, elevated cholesterol levels are not counterbalanced by sterol homeostatic mechanisms in the ER and cholesterol and other lipids continue to accumulate, causing the formation of abnormal lysosomal storage organelles. NPC disease is caused by mutations in NPC-1 and -2 proteins located in LE/LY that are believe to coordinate cholesterol egress from LE/LY, but the precise defect remains unknown. In addition to a role for NPC proteins, an underlying cause for cholesterol trafficking defects in NPC may be changes in the activity of proteins that regulate endosomal motility. LE/LY exhibit bidirectional motility between the periphery and the pericentriolar region of cells that is controlled in part by Rab GTPases. It has been shown that this motility is compromised in NPC cells and that overexpression of Rab 7 and 9 proteins reduce the NPC phenotype [3], [4]. Much is yet to be learned about cholesterol trafficking in general. Difficulty in the overall understanding of intracellular cholesterol movement arises from the fact that different mechanisms (vesicular and non-vesicular) operate simultaneously to move cholesterol [1], [2]. Therefore, further description of the protein and lipid factors that control intracellular cholesterol transport and content are important for a better understanding of cholesterol homeostasis.

We have previously performed a proteomic analysis of molecules that associated with detergent-resistant membranes (DRMs) [5]. This analysis allowed us to identify a novel protein whose mRNA is ubiquitously expressed. It binds membranes through N-terminal acylations, and possesses two canonical di-leucine signals involved in endosome targeting [6]. The protein was indeed mainly localized in LE/LY. Thus, we have named this protein Pdro for protein associated with DRMs and endosomes. While this manuscript was in preparation, two groups reported the characterization of the same protein. Nada *et al*, described the rodent orthologue of Pdro named p18 [7]. Similarly to Pdro, p18 was found to be anchored to DRMs and to localize to LE/LY. The authors reported that p18 plays an important role in endosome dynamics by recruiting through binding to p14-MP1, a scaffold for MEK1, the ERK pathway to LE. More recently, in a collaborative study, the authors also reported that the p18-p14-MP1 complex interacts with the Rag GTPases and is necessary for mammalian target of rapamycin complex 1 (mTORC1) activation by amino acids [8]. Hoshino *et al*, on the other hand, described the human protein named p27RF-Rho [9]. In contrast with our results and those from Nada *et al*, p27RF-Rho showed a punctate distribution that co-localized with punctate actin structure. p27RF-Rho was demonstrated to bind to p27^kip1^ and to regulate RhoA activation, a small GTPase that is a key regulator of the actin cytoskeleton [10]. The apparent discrepancy in cellular localization of Pdro and p18 on one hand, and p27RF-Rho on the other hand, is unclear at the moment. Thus, these results indicate a role for Pdro in endocytic trafficking, mTORC1 activation and actin cytoskeleton dynamics. Interestingly, the endocytic trafficking and the actin dynamics processes are connected, as the actin cytoskeleton is known to participate to LE/LY dynamics. Indeed, disrupting filamentous actin results in the redistribution of late endocytic organelles to the cell periphery, altered endosome motility and inhibition of cargo degradation [11], [12], [13], [14].

Herein we present evidence that cellular sterol levels regulate Pdro expression, which in turn affects both LDL-cholesterol uptake and cholesterol release from LE/LY that likely contribute to the altered intracellular free cholesterol level. We propose that the regulatory function of Pdro on cellular cholesterol homeostasis implicates LE/LY motility that is involved in LDL-derived cholesterol traffic.

## Results and Discussion

### Identification and Characterization of Pdro

**Figure 1:**
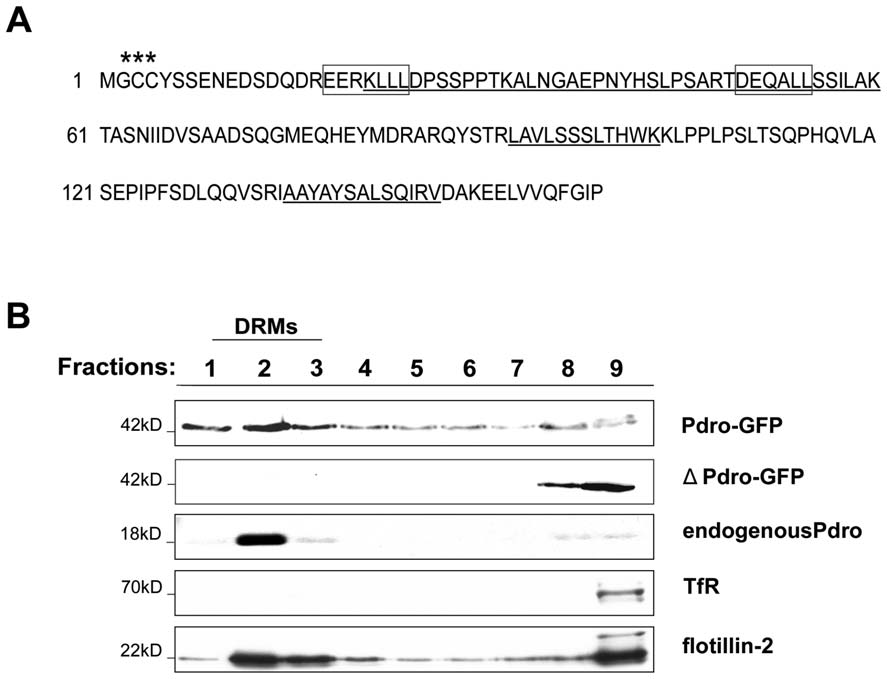
Identification and characterization of Pdro. (A) Full-length amino acid sequences of Pdro. Underlined amino acids indicate the position of peptide sequences determined by LC-MS/MS, boxes correspond to di-leucine motif (D/EXXXLL), stars indicate palmitoylation and myristoylation sites (C3 and 4 and G2 respectively). (B) DRMs from cells stably expressing Pdro-GFP (a c-terminus GFP-tagged protein) or ΔPdro-GFP (a c-terminus GFP-tagged mutant protein in which both Cys2 and 3 residues were substituted with Ala residues) were separated on a bottom-loaded sucrose step gradient. Probing the membrane with anti-GFP, anti-Pdro, anti-flotillin (DRMs marker) or anti-transferrin receptor (TfR, non-DRMs marker) antibodies assessed the distribution of proteins within fractions. Molecular–size standards (in kD) are shown on the left of the panel. The numbers represent the different fractions of the gradient.

DRMs from the human neuroblastoma cell line SHEP were purified as previously described [5] and resolved onto SDS-PAGE gels. A Coomassie-stained band (apparent molecular weight of 18 kD) excised from the gels was subjected to proteomic analysis using liquid chromatography and tandem mass spectrometry [5]. Five peptides were sequenced in MSMS mode and the protein identified corresponds to a protein of 161 amino acids, FLJ20625 (Figure 1A). According to HUGO Gene Nomenclature Committee, this protein is encoded by gene C11orf59 (chromosome11 open reading frame 59) located at 11q13.4. Complete open reading frame of the corresponding cDNA was obtained by RT-PCR from SHEP cells, using primers designed on the basis of the cDNA sequence available at GenBank/EMBL (accession number BC001706). The mRNA of Pdro was expressed in various human tissues (data not shown). A large-scale study reported that Pdro is myristoylated (Gly2) and palmitoylated (Cys3 and 4) at its N-terminus [15]. A C-terminus GFP-tagged mutant protein in which both Cys3 and 4 residues were substituted with Ala residues (ΔPdro-GFP) was no longer recovered from DRMs, as compared with a wild type GFP-tagged protein (Pdro-GFP) and the endogenous Pdro (Figure 1B). Thus, these modifications serve to localize Pdro to DRMs.

**Figure 2:**
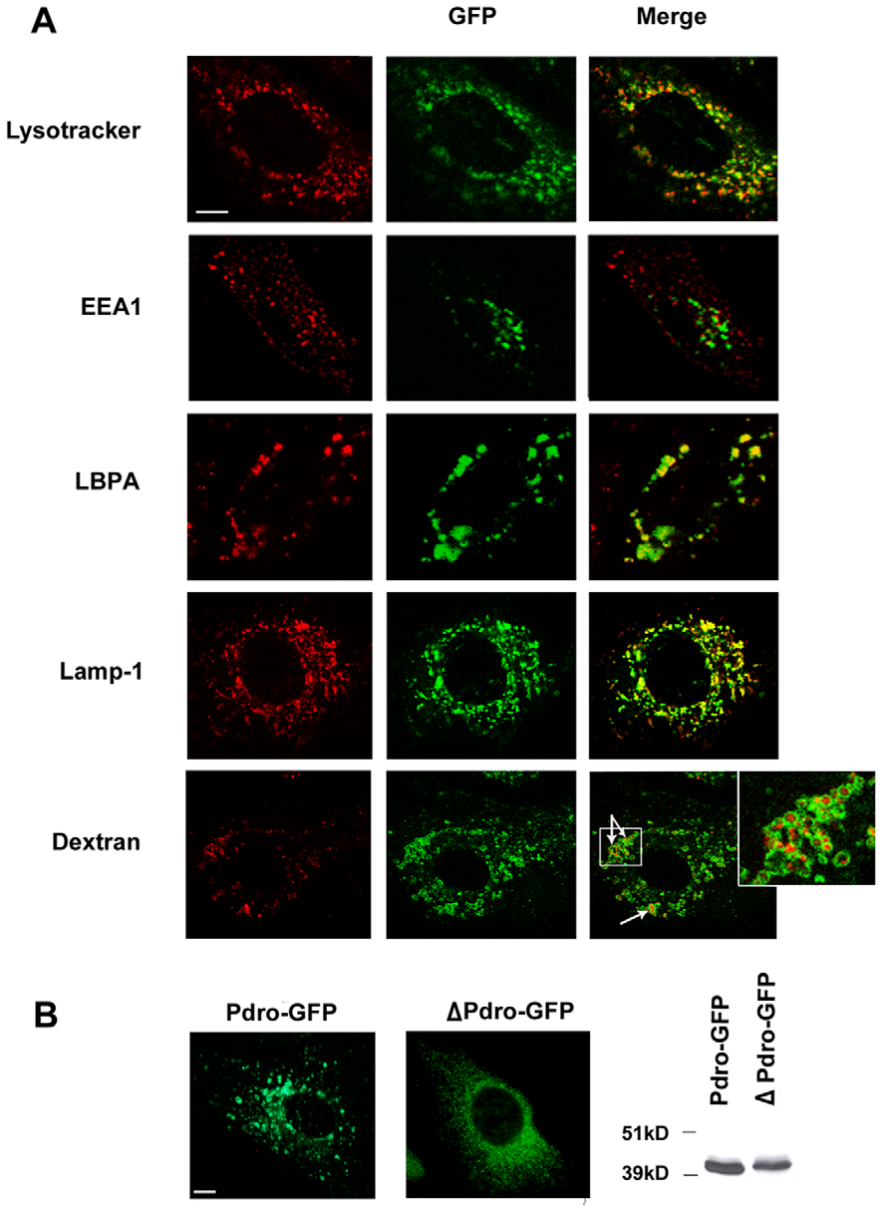
Intracellular localization of Pdro. (A) Pdro-GFP (green) expressing SHEP cells were either incubated with Lysotracker for 30 min and fixed before mounting, or fixed, permeabilized, and incubated with antibodies to EEA-1, LBPA and Lamp-1 to label endosomal compartments. For LE/LY labelling with Dextran, Pdro-GFP expressing SHEP cells were incubated with Alexa Fluor 488 Dextran. Co-distribution was assessed by confocal microscopy and is visualized in yellow in the merged images. Boxed areas are shown magnified. Scale bar = 10 µm. (B) Immunofluorescence images show intracellular localization of Pdro in SHEP cell lines stably expressing Pdro-GFP, and ΔPdro-GFP using antibodies directed to GFP. Scale bar = 10 µm. Cellular lysates from these cell lines were subjected to SDS-PAGE and immunoblotted with antibodies against GFP tag (Right panel). Molecular size standards (in kD) are shown on the left of the panel.

Very recently, the same human protein named p27RF-Rho and its rodent orthologue term p18 has been reported [9], [7], [8]. Intriguingly, p27RF-Rho was localized with punctate actin structure, whereas rodent p18 was localized to LE/LY. Pdro's sequence displays two canonical di-leucine signals between amino acids 17–22 and 49–54 (Figure 1A, boxed sequences) necessary for some proteins to be targeted to endosomes [6]. Accordingly, and consistent with rodent p18 localisation, our results show that Pdro is associated with the LE/LY compartment. Analysis by confocal microscopy of SHEP cells stably expressing GFP-tagged Pdro, showed an extensive co-localization of Pdro-GFP with LysoTracker Red-positive acidic vesicles (Figure 2A). The distribution of early endosomal autoantigen 1(EEA1)-positive endosomes did not match that of Pdro-GFP, whereas Pdro-stained vesicles colocalized substantially with lysobisphosphatidic acid (LBPA)-positive LE, and Lysosome-associated membrane protein 1 (LAMP-1)-positive LY. To substantiate further these findings, cells were allowed to internalize Alexa fluor 488-conjugated dextran for 2 hours to label LE/LY [16]. Dextran was found to co-localize with Pdro-labelled structures, which often appeared as ring-shaped vesicles that include dextran (Figure 2A, inset), indicating that Pdro associates with the membrane rather than with the lumen of the lysosomes. The localization of Pdro to LE/LY depends upon its association with DRMs as the Cys-mutant (ΔPdro-GFP) lost the perinuclear LE/LY punctate staining and appeared more dispersed throughout the cytoplasm (Figure 2B). Whether DRMs are related to lipid rafts remains unclear. They cannot be equated with native lipid domains because detergents can scramble lipids. Nevertheless, DRMs represent a more ordered membrane environment [17], suggesting that Pdro might tend to associate with particular lipid domain in LE/LY. Indeed, endosomes are thought to contain a mosaic of structural and functional domains [18]. The reason why p27RF-Rho was not found to localize to LE/LY is unclear at the moment.

### Cellular Sterol Levels Regulate Pdro Expression

**Figure 3:**
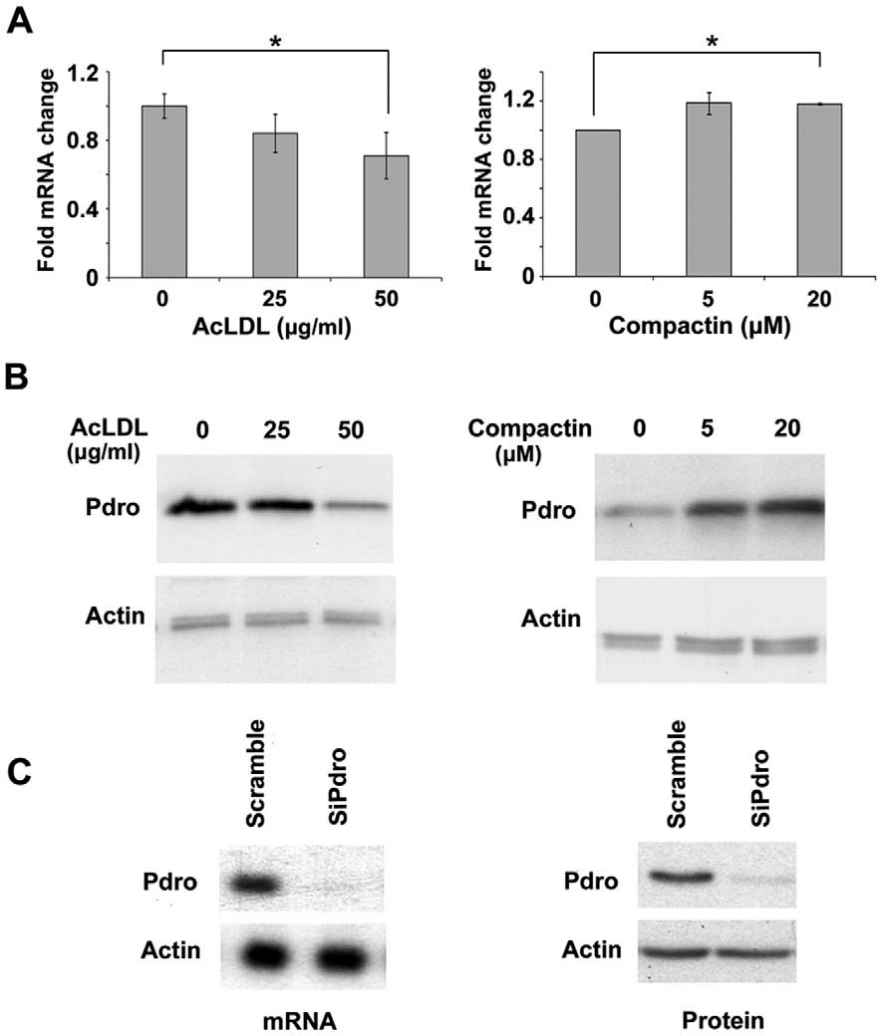
Cellular sterol levels regulate Pdro expression. (A) Manipulation of cellular cholesterol levels were performed by treating cells with the indicated AcLDL concentrations for 24h to stimulate cholesterol loading, or using the indicated concentrations of compactin for 24h to reduce cellular cholesterol. Pdro mRNA levels quantified by real-time PCR are expressed as relative units after internal normalization to TBP mRNA levels and compared with control samples in the absence of treatment. Values represent the mean ± SD of three independent experiments (*, p≤0.05 using Mann Whitney test). (B) Cellular lysates from these AcLDL or compactin treated cells were subjected to SDS-PAGE and immunoblotted with antibodies against endogenous Pdro. Actin served as loading controls. (C) SHEP cells were treated with siPdro or a control scramble siRNA (scramble). After 72h, cellular lysates were analyzed for Pdro mRNA content by Northern Blotting (mRNA) or for Pdro (protein) content by immunoblotting using anti-Pdro antibody. Actin served as loading controls.

Examination of the human Pdro promoter reveals that the 2kb region upstream the transcription start site contains five putative SRE motifs for SREBP-1/-2 binding (-1441/-1450; -1422/-1431; -926/-935; -208/-217 and -176/-185, respective to the transcription start site). Consistently, it has been recently reported that SREBPs occupy the promoter region of C11orf59 in a human hepatocyte cell line [19]. SRE are target sequences for the transcription factors SREBPs (SRE binding proteins) that regulate the transcription of numerous genes implicated in cholesterol homeostasis and fatty acid metabolism [20]. Therefore, Pdro may not only regulate endocytic trafficking and actin cytoskeleton dynamic, but it may also play a role in the regulatory circuitry of lipid homeostasis. Thus we tested whether manipulation of cellular cholesterol levels that control SREBPs activation [20] could alter the expression level of Pdro. Cholesterol loading was achieved by incubating cells with acetylated LDL (AcLDL) in the presence of lipoprotein-deficient serum [21]. We found that Pdro mRNA levels, monitored by real time RT-PCR, declined after LDL-cholesterol loading (Figure 3A). Pdro protein expression levels were also decreased upon LDL-cholesterol loading (Figure 3B). Cholesterol depletion was achieved by metabolic inhibition of cholesterol synthesis in the absence of supplemental sterol, using compactin [22]. Under these conditions, Pdro mRNA was slightly increased, whereas its protein expression was more clearly up-regulated (Figure 3B). This suggests that Pdro expression is further regulated at translational or post-translational level upon cholesterol depletion. Together, these results indicate that Pdro expression is regulated by cellular cholesterol levels. Precise understanding of the role of SREBPs in the regulation of C11orf59 expression is currently under investigation.

### Pdro Expression Level Alters Cellular Free Cholesterol Content

As mentioned earlier, mammalian cells growing under ordinary culture conditions derive their cholesterol preferentially from endocytic uptake of lipoproteins. Because Pdro localizes in LE/LY from which cholesterol derived from low-density lipoprotein egresses, we tested whether overexpression or depletion of Pdro could alter cellular free cholesterol content. We designed small interfering RNA (siPdro) that was able to substantially knock-down expression of endogenous Pdro (Figure 3C). Total cellular free cholesterol content was evaluated using flow cytometry analysis of filipin staining, a fluorescent marker for unesterified cholesterol. Our results demonstrated a ∼2 fold increase in cellular free cholesterol level in cells treated for 72 h with siPdro under ordinary culture conditions (Table 1). However, binding of FITC-wheat germ agglutinin lectin to cell surface glycoproteins and glycolipids remained unaltered, suggesting that Pdro depletion did not result in general cellular modification. To further substantiate the validity of the filipin staining analysis, the results from this technique were compared with those obtained by gas chromatographic analysis of free cholesterol content of cellular lysates. Both techniques yielded similar results (Table 1). Therefore, Pdro depletion increases cellular free cholesterol. Conversely, cells overexpressing a V5-tagged Pdro displayed a reduced level of cellular free cholesterol content, as compared to parental cells (SHEP: 12.3±3.42 µg/mg protein; SHEP-Pdro-V5: 3.91±1.21 µg/mg protein. Values are mean ± SD. n = 3, p≤0.05 using Mann Whitney test). This latter result also argues against an off-target effect of siPdro sequence. Finally, the effect of Pdro level on cholesterol was verified to be independent of cell confluence (data not shown). Thus, the expression levels of Pdro alter cellular free cholesterol content, indicating that Pdro participates in the regulatory circuitry of cholesterol homeostasis.

**Table 1:** Pdro knockdown increases cellular cholesterol content.

	Scramble	SiPdro
Filipin intensity (arbitrary units)^a^	432.33±1.61	**811.12±61.49** *
FITC-WGA fluorescence (arbitrary units)^a^	117.14±6.75	121.74±1.64
Free cholesterol (µg/mg protein)^b^	3.25±0.93	**6.27±1.48** *

a: Filipin and FITC-WGA labelling of the cells was quantified by flow cytometry (arbitrary units of fluorescence intensity).

b: Free cholesterol was quantified by gas chromatographic analysis after separation of cellular lipids by TLC. Values are mean ± SD of three independent experiments en triplicate wells.

*p≤0.05 using Mann Whitney test.

### Depletion of Pdro Increases Cholesterol Release from LE/LY

**Figure 4:**
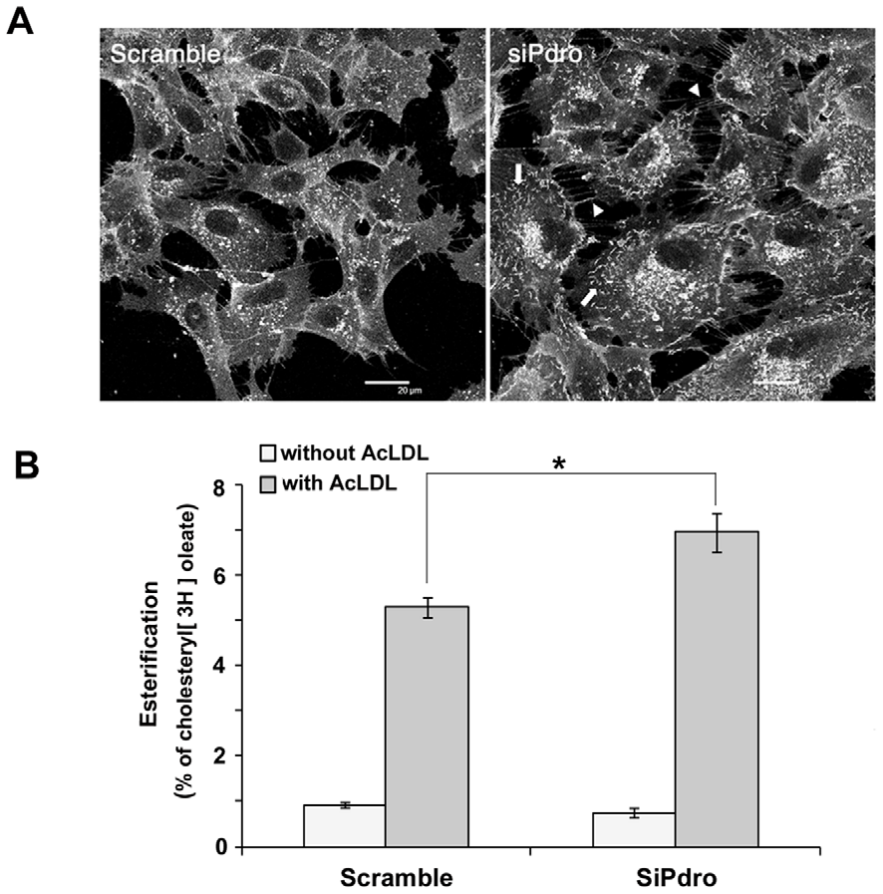
Pdro knockdown increases plasma membrane projections and cholesterol esterification. (A) Images of Filipin staining of SHEP cells treated for 72h with siPdro or scramble siRNA. Cholesterol-rich plasma membrane ruffles are indicated by arrows and filipodia by arrowheads. (B) Rate of cholesterol esterification in SHEP cells pre-treated for 72h with the indicated siRNA, followed by loading or not with 100 µg/ml AcLDL for 8h in the presence of [^3^H]oleate in lipoprotein-deficient serum. One representative experiment in triplicate wells is shown (*, p≤0.05 using Mann Whitney test).

The rodent orthologue of Pdro has been involved in controlling LE/LY dynamics [7], which in turn may affect LDL-derived cholesterol egress, as suggested in NPC disease [3], [4]. In this disease, the increased cellular free cholesterol content results form a lysosomal accumulation of LDL-derived cholesterol that causes the formation of abnormal lysosomal storage organelles [1]. Thus, we undertook confocal microscopy analysis of cellular filipin staining to investigate whether Pdro depletion has altered cellular free cholesterol distribution. In control cells, filipin staining was detected predominantly on perinuclear vesicular/granular network and on plasma membrane projections such as lamellipodia (Figure 4A). Visualization of cell surface membranous structures is likely facilitated by membrane convolution that generates an increase in fluorescence intensity [23]. This staining is consistent with the expected normal free cholesterol distribution, which includes enrichment in the plasma membrane, certain endosomes and part of the Golgi complex [2]. SiPdro-treated cells often appeared more spread than control-treated cells and they displayed an increase in filipin-stained membrane ruffles (arrow) and filopodia (arrow head). These membrane protrusions may result from elevated plasma membrane cholesterol content due to the increased cellular free cholesterol levels. Indeed, elevated membrane cholesterol causes an increase in membrane ruffling possibly through a misregulation of Rac GTPase [24]. They may also be a consequence of the depleted regulatory function of Pdro on RhoA GTPase activation [9], as the Rho GTPases are thought to exert mutual regulation to coordinate cytoskeletal behavior [25]. Nonetheless, neither an accumulation nor a decrease of cholesterol in the perinuclear network was readily observed. These data indicate that in contrast with NPC disease, Pdro depletion does not result in cholesterol accumulation in LE/LY.

We then asked whether the intracellular trafficking of the cholesterol released from LE/LY could be altered upon Pdro depletion. This released cholesterol moves to various destinations including the plasma membrane and the ER [26]. Esterification by the ER resident enzyme acyl-CoA∶cholesterol acyltranferase (ACAT) is often used as an assay for LDL-derived cholesterol to ER transport. It remains unclear however, whether most LDL-derived cholesterol transits through the plasma membrane before reaching the ER, or whether it can move directly to the ER [26]. To test whether Pdro depletion could interfere with cholesterol trafficking and correct delivery to the ER, we investigated cholesterol esterification by measuring incorporation of [^3^H]oleate into cholesteryl esters [27]. This was performed in lipoprotein-deficient serum in the presence or absence of 100 µg/ml of AcLDL, which is known to stimulate cholesterol esterification [28]. We found that in the absence of AcLDL, cellular cholesterol esterification rate was low in SHEP cells and only slightly affected following depletion of Pdro (Figure 4B). In contrast, in the presence of AcLDL, incorporation of [^3^H]oleate into cholesteryl esters was strongly increased. Upon Pdro depletion, AcLDL-stimulated cholesterol esterification was further augmented. In theory, this assay could measure esterification of both cellular and LDL cholesterol. However, the augmentation observed upon Pdro depletion mostly represents esterification of LDL-derived cholesterol, since in the absence of LDL (when only cellular cholesterol is present), cholesterol esterification was rather slightly diminished by the depletion of Pdro. These results strongly suggest that cholesterol flux between LE/LY and the ER is increased when Pdro is depleted. It is also consistent with the absence of cholesterol accumulation in the perinuclear network described in Figure 4A.

### Depletion of Pdro Stimulates Both LDL Uptake and Cholesterol Efflux from Cells

**Figure 5:**
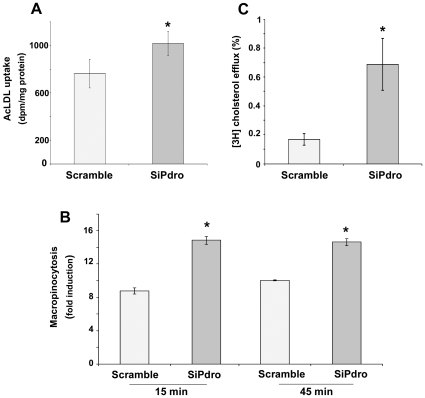
Pdro knockdown stimulates LDL uptake, macropinocytosis and cholesterol efflux. (A) For LDL uptake, cells pre-treated for 72h with the indicated siRNA and further incubated for 12h with [^3^H] cholesteryl oleate-labelled AcLDL (50 µg/ml) in lipoprotein lipoprotein-deficient serum. (B) For macropinocytosis determination, cells pre-treated for 72h with the indicated siRNA were incubated or not with RITC-dextran (2.5mg/ml) for various time, extensively washed and fixed with 4% PFA, and analyzed by flow cytometry. Values are compared with control samples in with RITC-dextran was omitted. (C) Cholesterol efflux to apoA-1 during 18h from SHEP was determined by incubating cells pre-treated for 72h with the indicated siRNA, with [^3^H] cholesterol and further incubation in serum-free medium containing 0.2% bovine serum albumin in the presence or absence of 10µg/ml apoA-I. Values are expressed as percentage of cholesterol efflux. Values represent the mean ± SD of three independent experiments (*, p≤0.05 using Mann Whitney test).

One mechanism that could contribute to increased cholesterol egress from LE/LY is an enhanced uptake of LDL. To test this hypothesis, control cells or cells depleted for Pdro were incubated with [^3^H]cholesteryl oleate-labeled LDL for 12 hours, and total radioactivity in cell homogenates was counted. As shown in Fig 5A, the uptake of LDL was significantly increased upon Pdro depletion, suggesting that the increase in free cellular cholesterol is due, at least in part, to an increased LDL uptake followed by an increased cholesterol egress from LE/LY. The LDL preparations used for uptake assay are modified LDL that are internalized through Scavenger Receptors or through non-receptor mediated fluid-phase macropinocytosis [29]. Considering that the plasma membrane protrusions described in Figure 4A are often associated with macropinocytic activity [30], we investigate whether Pdro knockdown could stimulate macropinocytosis. Control- or siPdro-treated cells were incubated with RITC-conjugated dextran and macropinocytic activity was analyzed by flow cytometry. Figure 5B shows that the amount of fluorescent dextran taken up by the cells was significantly increased upon knockdown of Pdro. Therefore, the absence of Pdro stimulates an increased macropinocytosis that may contribute to LDL uptake. The relative contribution of Scavenger Receptors and macropinocytosis in the increased LDL uptake is currently being investigated.

Excess cellular cholesterol is converted into nontoxic cholesteryl ester for storage or removed by cholesterol efflux at the plasma membrane [26]. In addition to the increased esterification depicted in Figure 4B, we examined whether cholesterol efflux was also increased upon Pdro knockdown. We therefore monitored the incorporation of [^3^H]cholesterol from radiolabeled cells into lipid-poor apoliprotein A-1 (apoA-1) [31]. ApoA-1 binds to the ubiquitously expressed ABC transporter A1, and triggers a multi-step process by which cholesterol is transferred from the plasma membrane to apoA-1 to generate disk-like high-density lipoprotein (HDL). Depletion of Pdro resulted in a clear increase in cholesterol efflux as compared with cells treated with control siRNA (Figure 5C). These data indicate that upon depletion of Pdro, the excess cholesterol is accompanied by an increase in cholesterol efflux from cells, possibly to prevent an over-accumulation of free cholesterol that is toxic to cells.

### Conclusion

Alterations in cholesterol and lipids contribute to certain diseases including atherosclerotic foam cells and Niemann-Pick type C disease. Recent progress in the understanding of the molecular basis of these disorders has arisen from the identification of proteins that control cholesterol transport and consequently affect cellular cholesterol content, such as Niemman-Pick Type C proteins or ABCA1 [1], [2]. In the present report, we reveal a role for the LE/LY-localized Pdro protein in the regulatory circuitry of cholesterol content. Cellular sterol levels regulate Pdro expression that in turn affects cellular cholesterol content. We revealed that Pdro depletion stimulated both LDL uptake and cholesterol release from LE/LY, providing a rationale to explain the increased free cellular cholesterol content observed upon Pdro knockdown. p18 has been reported to be essential for amino acid signalling to mTORC1 [8]. Whether an alteration of the mTORC1 signalling pathway could participate in the modulation of free cholesterol levels we describe here, is unclear at the moment and deserves further studies. On the other hand, the reported alteration in LE/LY dynamics upon p18 depletion could more directly participate in the increased LDL-derived cholesterol flux. p18 depletion results in the redistribution of LE/LY to the cell periphery [7]. It is noteworthy that depletion of p27-RF interferes with the formation of actin stress fibers [9], and that disruption of filamentous actin also results in LE/LY scattering [12], [13], [14]. Because LE/LY motility can affect cholesterol trafficking [3], [4], Pdro depletion might also alter LDL-derived cholesterol release independently of LDL uptake. Thus, by regulating the expression level of Pdro through their sterols levels, cells may engage a mechanism that regulates both LDL uptake and LE/LY dynamics, to ultimately control free cholesterol homeostasis.

## Materials and Methods

### Materials and Reagents

The antibodies used and their sources are as follow: Peroxidase-conjugated goat anti-mouse or anti-rabbit IgG, and Cy3 or Cy5-conjugated donkey anti-mouse IgG were from Jackson ImmunoResearch. Anti-LBPA antibody was a generous gift from Dr T. Kobayashi. The antibodies for EEA1 (clone 14) and Flotillin-2 (clone 29) were from BD Transduction Laboratories. The antibody for lamp-1 (clone H4A3) was from Developmental Studies Hybridoma Bank and for GFP from Roche Diagnostics. Lysotracker red DND-99, Alexa Fluor 488-labeled Dextran were from Molecular Probes. Filipin III was from Cayman chemicals. Geneticin, Lipofectamine 2000 were from Invitrogen. Rabbit anti-Pdro antibody and SiRNAs were developed by Eurogentec.

### Mass Spectrometry Identification

A discrete band was excised from the Coomassie blue-stained gel and in-gel digested as previously described [32]. Gel pieces were then extracted with 5% (v/v) formic acid solution and acetonitrile. After drying, tryptic peptides were resuspended in 0.5% aqueous trifluoroacetic acid. The samples were injected into a LC-Packings (Dionex) nanoLC system and first preconcentrated on a 300 µm×5 mm PepMap C18 precolumn. The peptides were then eluted onto a C18 column (75 µm×150 mm). The chromatographic separation used a gradient from solution A (5% acetonitrile: 95% water: 0.1% formic acid) to solution B (95% acetonitrile: 5% water: 0.1% formic acid) over 60 min at a flow rate of 300 nL×min^−1^. The LC system was directly coupled to a QTOF Ultima mass spectrometer (Waters). MS and MS/MS data were acquired and processed automatically using MassLynx 3.5 software. MS/MS data were processed using Mascot Distiller to produce peak lists that were subsequently submitted to the Mascot 1.8 (Matrix Science, UK) search engine for database search. Searches were performed against the NCBInr (20021102) database with the taxonomy “Mammalia”. Five distinct peptides corresponded to the Pdro protein. Four peptides have been identified with a score higher than 50 and the last one was checked manually.

### Transfection and Biochemistry Procedures

SHEP cells, maintained in RPMI (Sigma) containing 10% fetal bovine serum and antibiotics, were transfected (2 µg/ml of the plasmid) using Lipofectamine 2000 and selected using Geneticin (1mg/ml) for stable expression. SiRNA were transfected using Lipofectamine 2000 and 0.125nM siRNA duplexes: Si-Pdro, 5′-GGAGCUGGUUGUACAGUUU-3′; scramble siRNA was from Dharmacon. Cells were processed for immunoblotting, immunofluorescence or northern blotting 72h after transfection. Cellular lysates were done at 4°C using modified RIPA 1× (50 mM Tris-HCl pH 7.4, 1% NP40, 0.25% sodium deoxycholate, 150 mM NaCl, 1 mM EDTA, 1 mM NaOVO4, 1 mM NaF and protease inhibitors). Immunoprecipitation and Western blotting were performed as previously described [33] except that sodium deoxycholate was omitted from RIPA. DRMs purification was performed as described in [5].

### Cloning, Plasmids, Tissue Expression, Nothern Blotting and Real-Time RT-PCR

Complete open reading frame of the cDNA (GenBank/EMBL accession number BC001706, 1060bp cDNA, approximately 1300 bp mRNA), was obtained by RT-PCR from SHEP cells, using primers (5′-ACCATGGGGTGCTGCACAG-3′ and 5′-TGGGATCCCAAACTGTACAAC-3′), and cloned into pDONR221 or pcDNA-DEST47 (GFP tag) or pcDNA-DEST40 (V5 tag) following manufacturer's instructions (Gateway Technology, Invitrogen). Cys3 and 4 and Gly2 residues were mutated to alanines using QuickChange XL Site-directed mutagenesis Kit (Stratagene). Detection of Pdro mRNA expression in human tissues (Total RNAs from BD Biosciences) was done by RT-PCR using the above primers. GAPDH primers were used in the PCR as a control. Northern Blotting was performed as described by Anczukow et al. [34] using [^32^P] labeled cDNA of Pdro and actin as probes. For quantification of mRNAs by real-time RT-PCR, total RNA from SHEP cells was isolated using the Trizol reagent (Invitrogen, Courtaboeuf, France) according to manufacturer's instructions. First-strand cDNAs were synthesized from 500 ng of total RNA in the presence of 100 U of Superscript II (Invitrogen) and a mixture of random hexamers and oligo(dT) primers (Promega). Real-time PCR assays were performed with Rotor-GeneTM 6000 (Corbett Research, Mortlake, Australia). A list of the primers and real-time PCR assay conditions are available upon request (lefai@univ-lyon1.fr). The results were normalized using TBP (TATA binding protein) mRNA concentration, measured as reference gene in each sample.

### Immunofluorescence and Flow Cytometry Analysis

Immunostaining and dextran internalization was performed as described by Kauppi et al. [16]. Primary antibodies used to label endosomal compartment were revealed by incubation with Cy5-Conjugated anti-mouse secondary antibodies. Filipin staining of cholesterol was performed as described [35]. Cells were examined under a Leica TCS-SP confocal microscope equipped with an argon laser for UV excitations, and sections were constructed with Leica Confocal Software. Fluorescence signals were recorded sequentially using a 63× Plan-NEOFLUAR oil immersion objective. Images were processed for contrast and brightness using Photoshop (v8.0.1). Flow cytometry analysis of FITC-WGA were performed as described in [36] using a FACS Vantage SE (Becton Dickinson).

### Preparation of LDL and Determination of LDL Uptake and Macropinocytosis

Human LDLs were isolated from plasma by sequential ultracentrifugation. LDLs were acetylated with acetic anhydride by the method of Basu et al. [37]. Determination of LDL uptake was performed as previously decribed [27]. Briefly, LDLs were first labelled with [^3^H] cholesteryl oleate and re-isolated by ultracentrifugation before acetylation with acetic anhydride. Cells were then incubated for 12h with labelled-AcLDL in the presence of lipoprotein-deficient serum, lysed in 0.1% Triton and radioactivity determined by liquid scintillation counting. Total uptake was calculated as dpm/mg cell protein. For macropinocytosis quantification, cells were serum starved for 2 hours and incubated at 37°C in the presence of Rhodamine B-isothiocyanate (RITC)-dextran (2.5mg/ml) for various time. To terminate dextran uptake, cells were transferred to ice, washed three times with ice-cold PBS and fixed with 4% PFA (30 min at 4°C). Gently scrapped cells were resuspended in PBS and analysed by flow cytometry.

### Determination of Cholesterol Content, Cholesterol Esterification and Efflux

Cellular free cholesterol mass was determined as previously described [27]. Briefly, total lipids extracted from cell lysates were separated by thin layer chromatography. The silica gel containing free cholesterol was scraped, and analyzed by gas chromatography using an Econo-Cap EC-5 capillary column (30 m×0.32 µm, 0.25 µm) with helium as the carrier gas and quantified using stigmasterol as the internal standard. Free cholesterol determined by filipin intensity was performed by flow cytometry as described by Berger et al. [38]. Determination of cholesterol esterification was performed by quantification of [^3^H]oleate (0.5 µCi/ml) incorporation into cholesteryl ester for 8 hours in lipoprotein-deficient serum, in the presence or absence of AcLDL (100µg/ml), as previously described [27]. For determination of cholesterol efflux, cells were incubated in the presence of [^3^H] cholesterol (2µCi/ml) for 18h, washed three times with PBS and incubated in serum-free medium supplemented with 0.2% bovine serum albumin with or without 10µg/ml apoA-I for 18h. The medium and cells were collected separately, and radioactivity was determined by liquid scintillation counting. The percent cholesterol efflux was calculated as [^3^H]cholesterol counts in the medium divided by the sum of [^3^H] counts in the cell and in the medium.
